# Effects of airborne pollutants on mitochondrial DNA Methylation

**DOI:** 10.1186/1743-8977-10-18

**Published:** 2013-05-08

**Authors:** Hyang-Min Byun, Tommaso Panni, Valeria Motta, Lifang Hou, Francesco Nordio, Pietro Apostoli, Pier Alberto Bertazzi, Andrea A Baccarelli

**Affiliations:** 1 Laboratory of Environmental Epigenetics, Exposure Epidemiology and Risk Program, Harvard School of Public Health, Boston, MA, USA; 2 Department of Statistics, University of Milano-Bicocca, Milan, Italy; 3 Department of Environmental and Occupational Health, Università degli Studi di Milano and IRCCS Ca’ Granda Maggiore Policlinico Hospital, Milan, Italy; 4 Department of Preventive Medicine, Feinberg School of Medicine, Northwestern University, 680 N. Lakeshore Drive, Chicago 60611, USA; 5 Department of Experimental and Applied Medicine, Occupational Medicine and Industrial Hygiene, University of Brescia, Brescia, Italy; 6 Laboratory of Environmental Epigenetics, Exposure Epidemiology and Risk Program, Harvard School of Public Health, Room B12, 667 Huntington Avenue, Boston, MA 02115, USA

**Keywords:** Air pollutants, Mitochondria, DNA methylation

## Abstract

**Background:**

Mitochondria have small mitochondrial DNA (mtDNA) molecules independent from the nuclear DNA, a separate epigenetic machinery that generates mtDNA methylation, and are primary sources of oxidative-stress generation in response to exogenous environments. However, no study has yet investigated whether mitochondrial DNA methylation is sensitive to pro-oxidant environmental exposures.

**Methods:**

We sampled 40 male participants (20 high-, 20 low-exposure) from each of three studies on airborne pollutants, including investigations of steel workers exposed to metal-rich particulate matter (measured as PM_1_) in Brescia, Italy (Study 1); gas-station attendants exposed to air benzene in Milan, Italy (Study 2); and truck drivers exposed to traffic-derived Elemental Carbon (EC) in Beijing, China (Study 3). We have measured DNA methylation from buffy coats of the participants. We measured methylation by bisulfite-Pyrosequencing in three mtDNA regions, i.e., the transfer RNA phenylalanine (*MT-TF*)*,* 12S ribosomal RNA (*MT-RNR1*) gene and “D-loop” control region. All analyses were adjusted for age and smoking.

**Results:**

In Study 1, participants with high metal-rich PM_1_ exposure showed higher *MT-TF* and *MT-RNR1* methylation than low-exposed controls (difference = 1.41, *P* = 0.002); *MT-TF* and *MT-RNR1* methylation was significantly associated with PM_1_ exposure (beta = 1.35, *P* = 0.025); and *MT-RNR1* methylation was positively correlated with mtDNA copy number (r = 0.36; *P* = 0.02). D-loop methylation was not associated with PM_1_ exposure. We found no effects on mtDNA methylation from air benzene (Study 2) and traffic-derived EC exposure (Study 3).

**Conclusions:**

Mitochondrial *MT-TF* and *MT-RNR1* DNA methylation was associated with metal-rich PM_1_ exposure and mtDNA copy number. Our results suggest that locus-specific mtDNA methylation is correlated to selected exposures and mtDNA damage. Larger studies are needed to validate our observations.

## Background

Growing evidence from experimental models and human studies has shown that DNA methylation in the nuclear genome is susceptible to change in response to environmental exposures [1,2]. Epigenetic research on environmental exposures – such as airborne pollutants – that induce oxidative-stress in humans has consistently shown DNA methylation alterations in peripheral blood nuclear DNA [1,3-6]. DNA methylation has thus been proposed to reflect environmentally-induced epigenomic reprogramming and risk of future disease [7-9].

Mitochondrial dysfunction has been identified as an etiological determinant of a variety of human diseases. The vast majority of mitochondrial proteins are encoded in the nucleus and synthesized in the cytoplasm, but are imported into the mitochondria [10]. Mitochondrial DNA (mtDNA) is a small independent circular genome of 16.5 kb in humans. MtDNA includes genes encoding for 13 proteins, 22 transfer RNAs (tRNAs), and 2 ribosomal RNAs (rRNAs), which are synthesized by a separate mitochondrial translation system [11]. Mitochondria lack protective histones and have a relatively inefficient DNA repair system [12]. Therefore mtDNA is particularly vulnerable to reactive oxygen species (ROS), which are established determinants of DNA methylation alterations [13]. Exposure to airborne pollutants has been linked with increased mitochondrial DNA copy number [14], a marker of mtDNA damage, and alterations in mitochondrial gene expression [15].

Although mtDNA methylation was described more than three decades ago [16,17], methylation in the mtDNA genome has been inconsistently and rarely studied, mostly due to long-standing controversies about its functionality. In fact, up until the recent discovery of the mtDNA methyltransferase enzyme 1 (mtDNMT1) [18], mtDNA methylation did not appear to be actively regulated in mitochondria, thus hampering any conclusion about potential functions. Shock *et al*. demonstrated the presence of mtDNMT1 activity and cytosine methylation in mtDNA, as well as that mtDNMT1 translocates to mitochondria following a mitochondrion-targeting signal upstream of translational start sites [18]. MtDNMT1 gene expression is controlled by oxidative stress [18], a primary mediator of air pollution effects. Alteration of mtDNMT1 expression directly affects the transcription of mtDNA genes [18], thus confirming that mitochondrial gene transcripts are controlled by mtDNA methylation.

Despite these recent breakthroughs and widespread emerging interest in mtDNA methylation, whether environmental exposures influence epigenetic mechanisms in mtDNA has not yet been explored, particularly in human investigations. In the present study, we examined whether mtDNA methylation in three specific mtDNA loci, i.e., the *MT-TF*, *MT-RNR1* and displacement loop (D-loop) regions, differed in individuals with exposures to airborne pollutants. Mitochondrial tRNAs are essential for protein synthesis in mitochondria. Alteration in MT-TF is associated with several disease, such as neuromuscular disease [19], myoclonic epilepsy with ragged red fibers [20], mitochondrial myopathy [21], acute rhabdomyolysis [22], tubulointerstitial nephritis and stroke [23]. The *MT-RNR1* gene encodes for a protein that facilitates the formation of RNA secondary structures, assembly of the mitochondrial ribosome, and mitochondrial translation [24]. The D-loop region that we examined contains promoters for mtDNA transcription and nearly the entire mitochondrial genome transcribes from the D-loop [25]. We sampled groups with high- and low-exposure from three independent studies on different types of airborne pollutants including steel workers and controls exposed to metal-rich particulate matter (PM_1_) in Brescia, Italy (Study 1); gas-station attendants and controls exposed to traffic-derived air benzene in Milan, Italy (Study 2); and truck drivers and controls exposed to traffic-derived Elemental Carbon (EC) (Study 3) in Beijing, China. We also examined the association of mtDNA methylation with mtDNA copy number to add evidence to the functional significance of mtDNA methylation.

## Results

### Participant’s characteristics and exposure levels

Study 1 included workers from a steel-production plant in Brescia, Northern Italy, among whom we identified a high- and low-exposed group – each including 20 individuals – based on levels of exposure to metal-rich PM_1_. In the high-exposed steel workers, mean age was 42.5 ± 7.7 years, and 12 of them were ex/current smokers. In the controls, mean age was 37.6 ± 2.8 years, and 14 of them were ex/current smokers. The mean individual PM_1_ levels were 9.2 ± 2.4 μg/m^3^ (range: 7.6–11.8 μg/m^3^) in high-exposed steel workers and 2.5 ± 0.7 μg/m^3^ (range: 1.7–3.8 μg/m^3^) among controls (Table 1).

**Table 1:** Characteristics and exposure-levels of the study participants

**Study**		**Characteristics**	**High exposure**	**Low exposure**
**Study 1 Exposure to metal-rich particulate matter (PM)**			High-exposed steel workers (n = 20)	Controls (n = 20)
	Participants	Age [Years], mean ± SD	42.5 ± 7.7	37.6 ± 2.8
		Ex/current smokers, n (%)	12 (60)	14 (70)
	Exposure (PM_1,_ μg/m^3^)	Mean ± SD	9.2 ± 2.4	2.5 ± 0.7
		Range	[7.6; 11.8]	[1.7; 3.8]
**Study 2 Exposure to air benzene**			Gas-station attendants (n = 20)	Controls (n = 20)
	Participants	Age [Years], mean ± SD	39.9 ± 11.2	39.7 ± 10.4
		Ex/current smokers, n (%)	7 (35)	5 (25)
	Exposure (Benzene, μg/m^3^)	Mean ± SD	78.6 ± 42.5	7.0 ± 5.5
		Range	[31.2; 180.1]	[4.2; 23.0]
**Study 3 Exposure to traffic-derived elemental carbon**			Truck drivers (n = 20)	Controls (n = 20)
	Participants	Age [Years], mean ± SD	35.2 ± 5.1	33.4 ± 5.9
		Ex/current smokers, n (%)	8 (40)	6 (30)
	Exposure (Elemental Carbon, μg/m^3^)	Mean ± SD	21.3 ± 4.7	13.4 ± 2.1
		Range	[16.6; 35.6]	[7.8; 16.1]

SD: standard deviation.

Only male participants were selected in the present study.

Study 2 included 20 gas-station attendants with exposure to airborne benzene and 20 office workers as a control group. The mean age was 39.9 ± 11.2 in gas-station attendants and 39.7 ± 10.4 in office workers. Seven gas-station attendants and 5 controls were ex/current smokers. The mean personal benzene levels were 78.6 ± 42.5 μg/m^3^ (range: 31.2–180.1 μg/m^3^) in gas-station attendants and 7.0 ± 5.5 μg/m^3^ (range: 4.2–23.0 μg/m^3^) in controls (Table 1).

Study 3 included 20 truck drivers with exposure to traffic-derived air particles, measured as EC levels and 20 office workers as a control group, all living and working in Beijing, China. Mean age was 35.2 ± 5.1 years in truck drivers and 33.4 ± 5.9 in controls. Eight truck drivers and 6 controls were ex/current smokers. Mean individual EC exposure was 21.3 ± 4.7 μg/m^3^ (range 16.6–35.6 μg/m^3^) in truck drivers and 13.4 ± 2.1 μg/m^3^ (range: 7.8–16.1 μg/m^3^) in controls (Table 1).

All participants were healthy and did not report any chronic diseases at the time of recruitment.

### Levels of mtDNA methylation by group of exposure

**Figure 1:**
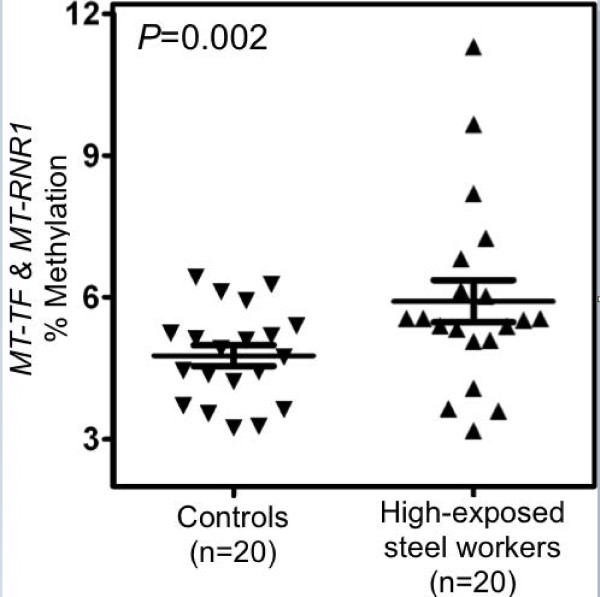
**Distribution of mitochondrial DNA methylation (%) in low-exposed controls and high-exposed steel workers.** The lines in the figure are mean and confidence intervals.

To determine the effect of environmental exposures on mtDNA methylation, we first contrasted high-exposure groups to controls in analysis adjusted for age and smoking (Table 2). In Study 1, *MT-TF* and *MT-RNR1* gene methylation was significantly higher in steel workers (6.47% ± 0.29) with high-exposure to PM_1_ exposure compared to controls (5.06% ± 0.30; *P* = 0.002). The distribution of *MT-TF* and *MT-RNR1* gene methylation among high-exposed steel workers and controls is shown in Figure 1. MtDNA methylation of the D-loop region was 2.16% ± 0.17 in high-exposed steel workers and 2.18% ± 0.17 in controls (*P* = 0.93) (Table 2). In Study 2 and Study 3, gas-station attendants and truck drivers did not show differences in DNA methylation compared to their respective control groups (Table 2).

**Table 2:** Mitochondrial DNA methylation level (%) by exposure groups of airborne pollutants

**Study 1**			
	**High-exposed steel workers (n** **=** **20)**	**Controls (n** **=** **20)**	**p-value**
***MT-TF *&*MT-RNR1***	6.47% ± 0.29	5.06% ± 0.30	0.002
**D-loop**	2.16% ± 0.17	2.18% ± 0.17	0.93
**Study 2**			
	Gas-station attendants (n = 20)	Controls (n = 20)	p-value
***MT-TF *&*MT-RNR1***	5.52% ± 0.22	5.89% ± 0.23	0.22
**D-loop**	1.68% ± 0.16	1.55% ± 0.16	0.53
**Study 3**			
	Truck drivers (n = 20)	Controls (n = 20)	p-value
***MT-TF *&*MT-RNR1***	5.37% ± 0.22	5.41% ± 0.22	0.88
**D-loop**	2.46% ± 0.54	2.38% ± 0.53	0.82

Data are presented as means ± standard deviations (SDs). Analysis adjusted for age and smoking status.

### Association of mtDNA methylation with exposure levels

We used multiple linear regression models adjusted for age and smoking to evaluate the association of mtDNA methylation with continuous levels of exposure to airborne pollutants in the three studies (Table 3). We calculated β coefficients to estimate the difference in mtDNA methylation between the 90th and 10th percentile of the distribution of exposure levels. In Study 1, PM_1_ levels were positively associated with *MT-TF* and *MT-RNR1* gene methylation (β = 1.35, SD = 0.33, and *P* = 0.025; Table 3). Mitochondrial D-loop region methylation was not associated with PM_1_ (β = −0.02, SD = 0.17; *P* = 0.96). We have also examined the association of mtDNA methylation with PM_2.5_, however it did not show a significant association (data not shown). In Study 2, air benzene levels were associated with neither *MT-TF* and *MT-RNR1* gene methylation (β = −0.37, SD = 0.08; *P* = 0.18) nor D-loop region methylation (β = −0.03, SD = 0.08; *P* = 0.91). In Study 3, EC exposure was associated with neither *MT-TF* and *MT-RNR1* gene methylation (β = −0.18, SD = 0.51; *P* = 0.64) nor mitochondrial D-loop region methylation (β = 0.56, SD = 0.85; *P* = 0.37).

**Table 3:** Associations of mitochondrial DNA methylation with exposure levels of airborne pollutants

**Marker**	β *^†^	SD*	p-value*
	**Study 1 – Association with metal-rich particulate matter (PM**_**1**_**)**		
***MT-TF *&*MT-RNR1***	1.35	0.33	0.025
**D-loop**	−0.02	0.17	0.96
	**Study 2 – Association with airborne benzene**		
***MT-TF *&*MT-RNR1***	−0.37	0.08	0.18
**D-loop**	−0.03	0.08	0.91
	**Study 3 – Association with traffic-derived elemental carbon**		
***MT-TF *&*MT-RNR1***	−0.18	0.51	0.64
**D-loop**	0.56	0.85	0.37

*Adjusted for age and smoking status. Exposure variables were log-transformed to improve model fit.

† Regression coefficient estimating the difference in mitochondrial DNA methylation (%) associated with an increase in exposure levels from the 90th to the10^th^ percentile.

### Sensitivity analyses

In sensitivity analyses, we repeated all statistical analyses after log-transforming the DNA methylation variables and found no major differences in the results (data not shown). We also conducted sensitivity analyses to exclude influences from potential differences in the proportion of blood leukocyte subtypes on the significant results observed in Study 1; multiple regression models that included percent granulocytes as independent variables – in addition to age and smoking – showed only negligible differences in the regression estimates for Study 1 and no changes in statistical significance (data not shown).

### Mitochondrial gene methylation and relative mitochondrial DNA copy number

**Figure 2:**
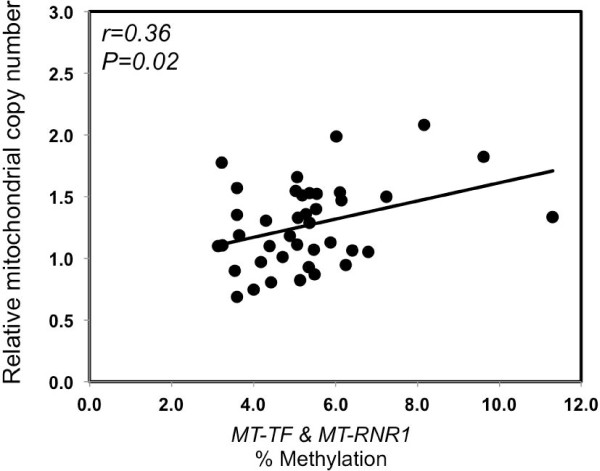
**Correlation between relative mitochondria copy number and mitochondrial gene *MT-TF *and *MT-RNR1 *methylation.**

To explore the functional significance of the association of *MT-TF* and *MT-RNR1* gene methylation with exposure to metal-rich PM_1_ in Study 1, we evaluated the correlation of mitochondrial *MT-TF* and *MT-RNR1* methylation with mtDNA copy number, a measure of damaged dysfunctional mitochondria. We obtained the mtDNA copy number by calculating the ratio between the estimated numbers of a mitochondrial gene and nuclear single copy gene, as previously reported for the same study [14]. As shown in Figure 2, individuals with higher *MT-TF* and *MT-RNR1* gene methylation exhibited higher mtDNA copy number (r = 0.36; *P* = 0.02).

## Discussion

In the present work, we measured DNA methylation at two mtDNA regions in individuals with exposures to three different types of air pollutants. We report, for the first time, associations of mtDNA methylation with exposure to air pollutants. Specifically, steel workers with high exposure to metal-rich PM exhibited higher levels of mitochondrial *MT-TF* and *MT-RNR1* gene methylation. Further, higher *MT-RNR1* gene methylation was significantly correlated with higher mtDNA copy number.

MtDNA methylation was first observed in 1984 in mice and reported to occur at very low level (<5%) using global measures of mtDNA methylation [26]. In the present work, we used a newly-developed quantitative and sensitive DNA methylation assays of mitochondrial *MT-RNR1* and D-loop methylation based on bisulfite-Pyrosequencing. Using this approach, we identified a subtle but significant difference in mtDNA methylation in steel workers exposed to metal-rich PM_1_ compared to controls. To the best of our knowledge, this finding represents the first report of a potential effect from environmental pollutants on mtDNA methylation in humans.

Mitochondria supply cellular energy by generating ATP through the respiration and oxidative phosphorylation (OXPHOS) system. The OXPHOS system is the primary intracellular source of ROS and free radicals under normal physiological and pathological conditions. Mitochondrial dysfunction may occur upon exposure to oxidative stress, and air pollutants are a well-established environmental source of cellular oxidative stress [27]. Microarray analyses have suggested that almost a quarter of the cellular responses to oxidative stress are mediated through mitochondrial dysfunction [28]. Van Houten *et al*. demonstrated that mtDNA accumulates 3–10 fold more damage than nuclear DNA after an oxidative-stress challenge [29]. PM is a major toxic component of air pollution that has been consistently associated in epidemiological investigations with increased incidence and mortality from respiratory disease [30], cardiovascular disease [14], and lung cancer [31]. We showed that mtDNA methylation in the *MT-TF* and *MT-RNR1* gene was specifically associated with metal-rich PM_1,_ whereas no association was found with airborne benzene or elemental carbon. Exposures pertaining to metal workers, who live in an environment with high oxidative potential, have been previously linked with mtDNA damage and mitochondrial dysfunction [32]. Taken together, these findings suggest that the chemical composition, source, and type of pollutant are critical to determine effects on mitochondrial DNA methylation. Future studies are warranted to evaluate the relative mitochondrial toxicity of metal-rich particles in larger prospective studies.

To explore the functional significance of the difference in *MT-TF* and *MT-RNR1* gene methylation associated with higher exposure to metal-rich PM in Study 1, we also examined the relationship between mitochondrial *MT-TF* and *MT-RNR1* methylation and mtDNA copy number and found a positive correlation between the two markers. Hobbie *et al*. have reported that a local conformational change in the 12S rRNA encoded by *MT-RNR1* sequence affects the efficiency and accuracy of codon–anticodon interactions [33]. The stem-loop structure of the 12S rRNA encoded by *MT-RNR1* is critical for normal function and integrity of the mitochondrial ribosome, and structural changes of 12S rRNA lead to decreased steady-state levels of 12S rRNA, instability of the small subunit of the ribosome, and abolished mitochondrial translation [24]. DNA methylation in the *MT-RNR1* gene may cause malfunction of mitochondrial ribosomes and abolished translation of mtDNA-encoded RNAs into proteins. Alternatively, increased mtDNA copy number in the presence of increased *MT-TF* and *MT-RNR1* gene methylation could simply represent a compensatory mechanism directed to maintain normal cellular function and cope with the increased respiratory demand required for ROS clearance [34].

We recognize a number of limitations in the present work. We measured mtDNA methylation markers in small samples of only 40 participants from each of the three studies. We cannot exclude that type II error due to limited statistical power might have led to the lack of associations with benzene exposure in Study 2 and EC in Study 3, as well as to the null findings for the mitochondrial D-loop region methylation data in Study 1. Our analysis of mtDNA methylation was limited to two circumscribed regions of the mitochondrial genome. However, we selected two regions with potential functional impacts, including a promoter region and a key ribosomal RNA sequence. Methods for extensive sequencing of the mitochondrial genome have not yet been developed and methylation assays for other mtDNA sequences are not readily available. Future work is warranted to determine whether DNA methylation in other regions on the mtDNA is specifically sensitive to air pollutants. Finally, although recent experimental data demonstrate that mtDNA methylation regulates gene expression, we did not measure RNA expression in our data due to the lack of suitable RNA samples for expression analysis. Therefore, we cannot determine the direct functional significance on RNA expression of the small differences in mtDNA methylation that we observed in the present work. It would also be interested to examine the association between nuclear genes, and particularly mtDNMT1 expression, with mtDNA methylation.

In summary, in the present work we found a moderate difference in mtDNA methylation of the *MT-TF* and *MT-RNR1* gene associated with exposure to metal rich PM. We did not find any difference in mtDNA methylation related to benzene and traffic-related EC level. Our data may serve as a preliminary conceptual model for larger future investigations of mtDNA methylation in human studies.

## Methods

### Study population

We sampled participants from three existing studies previously described in Dioni *et al*. [35] (Study 1); Bollati *et al*. [3] (Study 2); and Baccarelli *et al*. [36] (Study 3). From each of the three studies, we selected two groups of 20 high-exposed individuals and 20 low-exposed controls. In consideration of the predominance of males in the three studies and to limit potential confounding from gender, we only sampled male participants. In brief, from Study 1 we selected 20 individuals with high exposure to metal-rich particles (Particulate Matter with aerodynamic diameter >1 μm [PM_1_] ≥ 7.6 μg/m^3^) and 20 individuals with low exposure (PM_1_ ≤ 3.8 μg/m^3^) among workers in a steel production plant in Brescia, Italy. Personal PM_1_ levels were estimated using work-area measurements obtained through a GRIMM 1100 light-scattering dust analyzer (Grimm Technologies, Inc. Douglasville, GA, USA). From Study 2, we sampled 20 gas-station attendants with comparatively higher exposure to airborne benzene (personal airborne benzene ≥ 31.2 μg/m^3^) and 20 office workers with lower exposure (personal airborne benzene ≤ 23.0 μg/m^3^) in Milan, Italy. In all Study 2 participants, personal benzene exposure was determined by a passive sampler containing Chromosorb 106. From Study 3, we sampled 20 truck drivers in Beijing, China with high exposure to Elemental Carbon (EC ≥ 16.6 μg/m^3^), taken as a tracer of traffic particles. Age-matched controls were 20 office workers in Beijing, China with individual EC levels ≤ 16.1 μg/m^3^. In Study 3, we measured personal EC levels using active small-sized gravimetric samplers worn by the study participants during the eight hours on the day of the work, and for two consecutive days. We used standardized procedures for all studies to collect and store peripheral blood and extract DNA from buffy coat [3,35,36].

### DNA methylation analyses

We performed DNA methylation analysis with highly quantitative bisulfite-PCR pyrosequencing. Genomic DNA was treated with the EZ DNA methylation Gold kit (Zymo Research, Orange, CA, USA) according to the manufacturer’s instructions. Final elution volume was 40 μl with M-elution buffer. We designed the assay for the ‘MT-TF and MT-RNR1’ and mitochondrial ‘D-loop’ regions (primer sequences are shown in Additional file 1: Table S1). The ‘MT-TF and MT-RNR1’ assay interrogated two CpGs from MT-TF and MT-RNR1. The distance between the two CpGs was 83 base pairs, thus we took an average of two CpGs for MT-TF and MT-RNR1 gene methylation. The analysis by each gene is shown in Additional file 1: Table S2. We have measured 3 CpGs from D-loop region and took an average for the analysis. In order to exclude contamination from nuclear DNA in PCR amplification, we designed primer sequences unique for mtDNA sequences. Primer sequence is based on GenBank accession number J01415.2 which is shown in the L-strand of mitochondrial complete genome. A 30 μl of PCR was carried out using 15 μl of GoTaq Green Master mix (Promega), 10 pmol forward and 10 pmol reverse primers, 50 ng of bisulfite-treated DNA, and water to reach 30 μl final volume. PCR products were purified and sequenced by pyrosequencing as previously described using 0.3 μm sequencing primer [3]. Information for PCR primer is shown in Additional file 1. To monitor the intra- and inter- plate variation, we added universal genomic DNA (random genomic DNA) to each plate. We designed control oligo for 100% DNA methylation (PSQ-C oligo: 5′-TTGCGATACGACGGGAACAAACGTTGAATTC-3′) and 0% DNA methylation (PSQ-T oligo: 5′-TTGCGATACAACGGGAACAAACGTTGAATTC-3′). The sequencing primer for control oligo is 5′-AACGTTTGTTCCCGT-3′. We mixed PSQ-C oligo (or PSQ-T oligo) with sequencing oligo in PyroMark Annealing Buffer (QIAGEN Inc., Valencia, CA) and performed Pyrosequencing with sequencing entry C/TGTAT [37]. The methylation level is expressed using percent 5-methylcytosine (5mC). The correlation coefficient of the interplate variation was less than 1, which is considered low-variance. For example, the correlation coefficient of the D-loop assay in Study 1 was 0.56.

### Measurement of mtDNA copy number

Relative mtDNA copy number was measured by a quantitative real time polymerase chain reaction (Q-PCR) assay that measures the ratio of mitochondrial (Mt) copy number to single copy gene (S) copy number in experimental samples relative to a reference. The method is based on quantification of Mt and S quantities expressed as Cts derived from a standard curve obtained from serial dilutions of a reference DNA. A human beta-globin gene was used as reference single copy gene. The PCR primer and conditions are described in Hou L *et al.*[14]. Each run was completed by melting curve analysis to confirm the amplification specificity and absence of primer dimers. All samples were run in triplicates. For quality control purposes, 10 blind duplicate samples were interspersed among the test samples. The coefficient of variation for the Mt/S ratio in duplicate samples was 3.2%.

### Statistical analysis

We used standard descriptive statistics to present the study participants’ characteristics. Because DNA methylation results were obtained from two replicates that generated values for two or three CpG positions for *MT-TF* and *MT-RNR1* or D-loop, respectively, we used mixed-effects regression models [7] to evaluate the average associations of each mtDNA methylation marker with exposure group (high vs. low) or exposure levels (continuous variables, log-transformed to achieve higher estimate precision), by adapting the methods described by Boeke *et al.*[38]. We checked regression assumptions by performing diagnostic tests for each model, including the Shapiro-Wilk test for normality of residuals and White test for homogeneity of variance of residuals. All models were adjusted for age and smoking. A two-sided *P* <0.05 was considered statistically significant. All statistical analyses were performed in SAS (version 9.2; SAS Institute Inc., Cary, NC, USA).

## Competing interests

The authors declare that they have no competing interests.

## Authors’ contributions

HMB and AAB designed the experiment. TP and FN analyzed the data. HMB and VM performed the experiments. LF, PA, PAB, and AAB contributed participant’s materials. HMB and AAB wrote the manuscript and AAB oversaw the research. All authors have read and approved the final manuscript.

## Funding

This work was supported by funding from the National Institute of Environmental Health Sciences (ES000002; R01ES020268; R21ES020010; and R21ES019773).

## Supplementary Material

Additional file 1: Table S1 Bisulfite-Pyrosequencing primer sequence information. **Table S2**. Associations of mitochondrial DNA methylation with exposure levels of airborne pollutants. Click here for file
