# Supplementary material for: Effects of airborne pollutants on mitochondrial DNA Methylation
Source: Part Fibre Toxicol. 2013 May 8;10:18. doi: 10.1186/1743-8977-10-18 (PMC3660297; doi:10.1186/1743-8977-10-18)
Supplement: Additional file 1: Table S1 — Bisulfite-Pyrosequencing primer sequence information. Table S2. Associations of mitochondrial DNA methylation with exposure levels of airborne pollutants. [file 1743-8977-10-18-S1.docx]

Additional file. Table 1 Bisulfite-Pyrosequencing primer sequence information

| Primer | Sequence | Mt genome location (np) | Amplicon size (bp) | Annealing temperature (^o^C) | Target CpGs |
| --- | --- | --- | --- | --- | --- |
| MT-TF and MT-RNR1-F | 5’-TAAAGTAATATATTGAAAATGTTTAGA-3’ | 599-621 | 168 | 54 | 2 CpGs |
| MT-TF and MT-RNR1-R(bio) | 5’-TACTTAATACTTATCCCTTTTAATC-3’ | 742-766 |  |  |  |
| MT-TF and MT-RNR1-sp | 5’-TATTGAAAATGTTTA-3’ | 609-625 |  |  |  |
| D-loop-F | 5’-TGTGTAGATATTTAATTGTTATTA-3’ | 236-259 | 254 | 54 | 3 CpGs |
| D-loop-R(bio) | 5’-CAAATCTATCACCCTATTAACCAC-3’ | 6-29 |  |  |  |
| D-loop-sp | 5’-TAATTAATTAATATATTT-3’ | 200-218 |  |  |  |

Additional file. Table 2 Associations of mitochondrial DNA methylation with exposure levels of airborne pollutants

| **Effect** | **Study** | **CpG Position** | **β** *^†^ | **SD*** | **p-value*** |
| --- | --- | --- | --- | --- | --- |
| High exposer vs. Controls | Study 1  Exposure to metal-rich particulate matter (PM_1_) | 1 (*MT-TF*) | 0.43 | 0.33 | 0.193 |
|  |  | 2 (*MT-RNR1*) | 0.51 | 0.29 | 0.085 |
|  | Study 2  Exposure to air benzene | 1 (*MT-TF*) | 0.33 | 0.26 | 0.203 |
|  |  | 2 (*MT-RNR1*) | -0.51 | 0.26 | 0.058 |
|  | Study 3  Exposure to traffic-derived elemental carbon | 1 (*MT-TF*) | -0.33 | 0.27 | 0.228 |
|  |  | 2 (*MT-RNR1*) | 0.13 | 0.26 | 0.626 |

*Adjusted for age and smoking status. Exposure variables were log-transformed to improve model fit.

† Regression coefficient estimating the difference in mitochondrial DNA methylation (%) associated with an increase in exposure levels from the 90^th^ to the10^th^ percentile.
